# EasyClone: method for iterative chromosomal integration of multiple genes in *Saccharomyces cerevisiae*

**DOI:** 10.1111/1567-1364.12118

**Published:** 2013-11-18

**Authors:** Niels B Jensen, Tomas Strucko, Kanchana R Kildegaard, Florian David, Jérôme Maury, Uffe H Mortensen, Jochen Forster, Jens Nielsen, Irina Borodina

**Affiliations:** 1 The Novo Nordisk Foundation Center for Biosustainability, Technical University of DenmarkHørsholm, Denmark; 2 Department of Systems Biology, Technical University of DenmarkKgs Lyngby, Denmark; 3 Department of Chemical and Biological Engineering, Chalmers University of TechnologyGothenburg, Sweden; 4 Evolva Biotech A/S, lersø parkallé 42-44, DK-2100København Ø, Denmark

**Keywords:** genome editing, *Saccharomyces cerevisiae*, metabolic engineering, integrative vectors, USER cloning

## Abstract

Development of strains for efficient production of chemicals and pharmaceuticals requires multiple rounds of genetic engineering. In this study, we describe construction and characterization of EasyClone vector set for baker's yeast *Saccharomyces cerevisiae*, which enables simultaneous expression of multiple genes with an option of recycling selection markers. The vectors combine the advantage of efficient uracil excision reaction-based cloning and Cre-LoxP-mediated marker recycling system. The episomal and integrative vector sets were tested by inserting genes encoding cyan, yellow, and red fluorescent proteins into separate vectors and analyzing for co-expression of proteins by flow cytometry. Cells expressing genes encoding for the three fluorescent proteins from three integrations exhibited a much higher level of simultaneous expression than cells producing fluorescent proteins encoded on episomal plasmids, where correspondingly 95% and 6% of the cells were within a fluorescence interval of Log_10_ mean ± 15% for all three colors. We demonstrate that selective markers can be simultaneously removed using Cre-mediated recombination and all the integrated heterologous genes remain in the chromosome and show unchanged expression levels. Hence, this system is suitable for metabolic engineering in yeast where multiple rounds of gene introduction and marker recycling can be carried out.

## Introduction

Baker's yeast *Saccharomyces cerevisiae* is an attractive cell factory for industrial biotechnology (Kim *et al*., 2012). It is used for the production of food and beverages, and for chemicals, enzymes and pharmaceuticals. Due to extensive efforts within yeast genetic research, a vast number of genetic and molecular tools have been developed [for reviews see (Da Silva & Srikrishnan, 2012; Krivoruchko *et al*., 2011; Siddiqui *et al*., 2012)]. Among these tools, high and low copy as well as integrative plasmids have found extensive use in gene function studies and in metabolic engineering (Hong & Nielsen, 2012). There are strengths and weaknesses for each type of plasmids and eventually the choice depends on the overall goal. When it comes to metabolic engineering of yeast to obtain a process with high titer, rate, and yield, multiple rounds of strain engineering are commonly required. To cut down the costs, it is important that the turnaround time of the metabolic engineering cycle is as short as possible. At the same time, it is critical that the cycle is at the highest possible standard, for example, in terms of stability of expression of the genes introduced and that expression levels can be controlled in a reliable way either by inducible promoters or constitutive promoters of various strengths (see Da Silva & Srikrishnan, 2012, for references and details). Another important consideration for pathway engineering is the ability to co-express the introduced genes at the desired levels in each and every cell of the yeast population. This is a problem as the copy number, for both the high and low copy number plasmids, fluctuates in the cell population (Futcher & Carbon, 1986; Mead *et al*., 1986; Borodina *et al*., 2010). The stability issue can be overcome using integration plasmids, where the expression cassettes are integrated in the genome. Several integration vector series have been developed over time (Gietz & Akio, 1988; Sikorski & Hieter, 1989; Alberti *et al*., 2007; Sadowski *et al*., 2007). Despite the stable nature of chromosomal integrations when compared with for example, high copy episomal plasmids, instability can occur if the introduced fragments share a high degree of sequence homology or if insertions are multiple tandem insertions. If the latter is the case, there is a high risk of chromosomal rearrangements including loss of the introduced genes due to direct repeat recombination (Wang *et al*., 1996; Lee & Silva, 1997).

Another crucial step for the turnaround time of a metabolic engineering cycle is the cloning phase. Several high-throughput cloning methods developed over time have proven to be of great importance, for example Gateway™ cloning (Invitrogen) (Hartley *et al*., 2000), Gibson Assembly® cloning (New England Biolabs) (Gibson *et al*., 2009), Golden Gate cloning (Engler *et al*., 2008), and Infusion cloning from Clontech. Another method is the uracil-specific excision reaction (USER)-based cloning technique (Nour-Eldin *et al*., 2006). This cloning technique was the basic technique for the plasmid set developed by Mikkelsen *et al*. (2012), which allows for stable integration into 15 individual integration sites, where each site was validated for growth impairment and expression of galactosidase. Furthermore, the insertion sites on each chromosome are interspaced by essential genetic elements preventing loop out of the inserted fragments by homologous recombination. As an example of their system's applicability, the authors showed successful expression of a complex eight gene indole glucosinolate biosynthetic pathway in *S. cerevisiae*. One limitation of this plasmid set is the fact that it is based on only one selectable marker, *Kluyveromyces lactis URA3*, which needs to be recycled during sequential integration steps in a process mediated by direct repeat recombination and 5-fluoroorotic acid selection. Hence, introduction of multigene pathways will be time-consuming as it will require many rounds of strain transformation and marker elimination.

Our intention has been to create a method that allows repeated cycles of genetic engineering, in which multiple genes are simultaneously stably integrated into the genome of *S. cerevisiae*. We describe integrative vector set EasyClone with a wide repertoire of LoxP-flanked selection markers, developed on the basis of Mikkelsen *et al*. (2012) vectors. As a proof of concept, we simultaneously integrate three different gene targeting cassettes containing genes encoding three different fluorescent proteins and then loop out the markers without losing fluorescent protein genes. We also evaluate the heterogeneity in the population of cells expressing multiple proteins from the integrative EasyClone vectors and from 2μ-based episomal plasmids.

## Materials and methods

### Strains and media

*Saccharomyces cerevisiae* CEN.PK102-5B (MAT*a ura3-52 his3Δ1 leu2-3/112 MAL2-8*^*c*^ *SUC2*) strain was obtained from Verena Siewers (Chalmers University). Yeast transformants were selected on synthetic complete (SC) drop-out media lacking the amino acids matching the auxotrophic markers on the plasmids used. These SC plates were made from premixed drop-out powders from Sigma-Aldrich. When yeast was grown in liquid media, it was either in SC, Delft, or standard yeast peptone dextrose (YPD) media. Delft contained (L^−1^): 7.5 g (NH_4_)_2_SO_4_, 14.4 g KH_2_PO_4_, 0.5 g MgSO_4_·7H_2_O, 22 g dextrose, 2 mL trace metals solution, and 1 mL vitamins. The pH of Delft medium was adjusted to 6 prior to autoclavation. Vitamin solution was added to Delft medium after autoclavation. Vitamin solution was added after autoclavation. The trace metals solution contained (L^−1^): 4.5 g CaCl_2_·2H_2_O, 4.5 g ZnSO_4_·7H_2_O, 3 g FeSO_4_·7H_2_O, 1 g H_3_BO_3_, 1 g MnCl_2_·4H_2_O, 0.4 g Na_2_MoO_4_·2H_2_O, 0.3 g CoCl_2_·6H_2_O, 0.1 g CuSO_4_·5H_2_O, 0.1 g KI, 15 g EDTA. The trace metals solution was prepared by dissolving all the components except EDTA in 900 mL ultra-pure water at pH 6. The solution was then gently heated and EDTA was added. In the end, the pH was adjusted to 4, and the solution volume was adjusted to 1 L and autoclaved (121 °C in 20 min). This solution was stored at + 4 °C. The vitamin solution had (L^−1^): 50 mg biotin, 200 mg p-aminobenzoic acid, 1 g nicotinic acid, 1 g Ca-pantothenate, 1 g pyridoxine-HCl, 1 g thiamine-HCl, 25 g myo-inositol. Biotin was dissolved in 20 mL 0.1 M NaOH and 900 mL water is added. pH was adjusted to 6.5 with HCl and the rest of the vitamins were added. pH was re-adjusted to 6.5 just before and after adding m-inositol. The final volume was adjusted to 1 L and sterile-filtered before storage at + 4 °C.

All standard cloning was carried out using *Escherichia coli* strain DH5α, which was grown in standard Luria–Bertani (LB) medium containing 100 μg mL^−1^ ampicillin. For the cloning of plasmid carrying the *ccdB* gene and chloramphenicol cassette, *E. coli ccdB* strain was used as a host strain and transformants were selected on LB medium containing 100 μg mL^−1^ ampicillin and 25 μg mL^−1^ chloramphenicol.

### Plasmids and strains construction

The episomal plasmids were generated as follows: the 1.8-kb fragment carrying the USER cassette, *ccdB* gene, and chloramphenicol marker was generated by PCR amplification using primers pESC_U_ccdB_fw and pESC_U_ccdB_rv and plasmid pCfB49 (pXII-1-ccdB) as a template. The PCR fragment was digested with SacI and XhoI, gel-purified, and then ligated into plasmid pESC-URA or pESC-HIS, which were digested with the same enzyme pair. The final plasmids were designated as pCfB54 (pESC-URA-ccdB-USER) and pCfB55 (pESC-HIS-ccdB-USER), respectively. Finally, the pCfB54 and pCfB55 plasmids were digested with FastDigest® AsiSI to remove the *ccdB* gene including the chloramphenicol marker and re-ligated to generate the final plasmids pCfB132 (pESC-URA-USER) and pCfB291 (pESC-HIS-USER).

To construct pCfB220 (pESC-LEU-USER), the 36-bp fragment carrying the USER cassette was excised from the plasmid pCfB132 (pESC-URA-USER) using SacI and XhoI, gel-purified, and then ligated into plasmid pESC-LEU, which was digested with the same enzyme pair.

The integration plasmids were made from the plasmid set previously described in Mikkelsen *et al*. (2012) by replacing the directed repeats (DR) flanked *K. lactis URA3* selection marker with different selection markers flanked with *LoxP* sites (Gueldener *et al*., 2002; Ito-Harashima & McCusker, 2004). The selection marker exchange was accomplished by uracil-specific excision reaction (USER) (Nour-Eldin *et al*., 2006), where the parent plasmids and the different *LoxP*-flanked selection marker fragments were PCR-amplified by PfuX7 polymerase (Nørholm, 2010) using oligos listed in Table1. The integration plasmids listed in Table2 were made by combining plasmid and selection markers’ PCR products as indicated in Table2 using the following protocol: 3 μL of gel-purified plasmid PCR product was mixed with 5 μL of gel-purified selection marker PCR fragment together with 1 μL Taq polymerase buffer and 1 μL USER enzyme (NEB). The mix was incubated at 37 °C for 25 min, at 25 °C for 25 min and transformed into chemically competent *E. coli* DH5α. The clones with correct inserts were identified by colony PCR, and the plasmids were isolated from overnight *E. coli* cultures and confirmed by sequencing. This way the following plasmids were obtained: pCfB255, pCfB257, pCfB258, pCfB259, pCfB260, pCfB261, pCfB262, pCfB353, pCfB388, pCfB389, pCfB390, pCfB391.

**Table 1:** List of the primers used for vector construction and strain verification. USER-specific overhangs are marked in bold, translational enhancer (Kozak) sequence is underlined (Cavener & Ray, 1991; Nakagawa *et al*., 2008)

Name	Sequence	Application
pESC_U_ccdB_fw	5′-AAAAGAGCTCGAATGCGTGCGATCGCAG-3′	Amplification of USER cassette, *ccdB* gene, and chloramphenicol cassette
pESC_U_ccdB_rv	5′-AAAACTCGAGGAATGCACGCGATCGCTG-3′	
ID399USERrev	5′-**ATTGGGU**GCATAGGCCACTAGTGGATCTG-3′	Amplification of *LoxP*-flanked selection marker cassettes
ID400USERfwd	5′-**ATCGCGU**CAGCTGAAGCTTCGTACGC-3′	
ID401pIntFwdU	5′-**ACCCAAU**TCGCCCTATAGTGAGTCG-3′	Amplification of integrative plasmid backbone
ID402pintRevU	5′-**ACGCGAU**CTTCGAGCGTCCCAAAACC-3′	
ID1493	5′- **CGTGCGAU**CCGCATAGGGAGTGTAAATTTATC -3′	Amplification of positive GFP control fragment for USER plasmid verification
ID1494	5′-**CACGCGAU**AGTGAAAGGAAGGCCCATGAG -3′	
PTEF1_fw	5′-**ACCTGCACU**TTGTAATTAAAACTTAG-3′	Amplification of *TEF1* promoter
PTEF1_rv	5′-**CACGCGAU**GCACACACCATAGCTTC-3′	
YFP/CFP_F+	5′-**AGTGCAGGU**AAAACAATGAGTAAAGGAGAAGAACTTTTCAC-3′	Amplification of *YFP* and *CFP* genes
YFP/CFP_R+	5′-**CGTGCGAU**TCATTTGTATAGTTCATCCATGCCATG-3′	
RFP_F+	5′-**AGTGCAGGU**AAAACAATGGCCTCCTCCGAGGACGTCATC-3′	Amplification of *RFP* gene
RFP_R+	5′-**CGTGCGAU**TCAGGCGCCGGTGGAGTGGCGG-3′	
ID901 X-2-up-out	5′-TGCGACAGAAGAAAGGGAAG-3′	PCR with ID339 verifies insertion in X-2-UP
ID902-X-2-down-out	5′-GAGAACGAGAGGACCCAACAT-3′	PCR with ID401 verifies insertion in X-2-DW
ID903-X-3-up-out	5′-TGACGAATCGTTAGGCACAG-3′	PCR with ID339 verifies insertion in X-3-UP
ID904-X-3-down-out	5′-CCGTGCAATACCAAAATCG-3′	PCR with ID401 verifies insertion in X-3-DW
ID905-X-4-up-out	5′-CTCACAAAGGGACGAATCCT-3′	PCR with ID339 verifies insertion in X-4-UP
ID906-X-4-down-out	5′-GACGGTACGTTGACCAGAG-3′	PCR with ID401 verifies insertion in X-4-DW
ID339-TEF1_test_rv	5′-GCTCATTAGAAAGAAAGCATAGC-3′	Verification of insertion of constructs containing *TEF1*

**Table 2:** List of plasmids used in this study

Name	Description	Reference
Integrative plasmid set with *URA3* selection marker flanked with direct repeats		
pCfB126	pX-2-USER-URA3-DR	Mikkelsen *et al*. (2012)
pCfB127	pX-3-USER-URA3-DR	Mikkelsen *et al*. (2012)
pCfB128	pX-4-USER-URA3-DR	Mikkelsen *et al*. (2012)
pCfB383	pXI-1-USER-URA3-DR	Mikkelsen *et al*. (2012)
pCfB384	pXI-2-USER-URA3-DR	Mikkelsen *et al*. (2012)
pCfB385	pXI-3-USER-URA3-DR	Mikkelsen *et al*. (2012)
pCfB387	pXI-5-USER-URA3-DR	Mikkelsen *et al*. (2012)
pCfB129	pXII-1-USER-URA3-DR	Mikkelsen *et al*. (2012)
pCfB120	pXII-2-USER-URA3-DR	Mikkelsen *et al*. (2012)
pCfB130	pXII-4-USER-URA3-DR	Mikkelsen *et al*. (2012)
pCfB131	pXII-5-USER-URA3-DR	Mikkelsen *et al*. (2012)
pCfB49	pXII-1-ccdB-USER-URA3-DR	Mikkelsen *et al*. (2012)
Plasmids that contain LoxP-flanked selection marker cassettes		
pUG6	LoxP-KanMX	Gueldener *et al*. (2002)
pUG27	LoxP-SpHIS5	Gueldener *et al*. (2002)
pUG72	LoxP-KlURA3	Gueldener *et al*. (2002)
pUG73	LoxP-KlLEU2	Gueldener *et al*. (2002)
pSA40	LoxP-CaLYS5	Ito-Harashima & McCusker (2004)
Episomal replication vectors with USER cassette (the vectors were derived from pESC vector series, Agilent)		
pCfB54	pESC-URA-ccdB-USER	This study
pCfB55	pESC-HIS-ccdB-USER	This study
pCfB132	pESC-URA-USER	This study
pCfB291	pESC-HIS-USER	This study
pCfB220	pESC-LEU-USER	This study
EasyClone integrative vector set with loxP-flanked selection markers		
pCfB255	pX-2-LoxP-KlURA3	This study
pCfB353	pX-2-LoxP-KanMX	This study
pCfB257	pX-3-LoxP-KlLEU2	This study
pCfB258	pX-4-LoxP-SpHIS5	This study
pCfB388	pXI-1-LoxP-KlLEU2	This study
pCfB389	pXI-2-LoxP-KlURA3	This study
pCfB390	pXI-3-LoxP-KlURA3	This study
pCfB391	pXI-5-LoxP-SpHIS5	This study
pCfB259	pXII-1-LoxP-KlLEU2	This study
pCfB260	pXII-2-LoxP-CaLYS5	This study
pCfB262	pXII-4-LoxP-SpHIS5	This study
pCfB261	pXII-5-LoxP-SpHIS5	This study
Plasmids containing genes encoding fluorescent proteins		
pWJ1163	CFP	Reid *et al*. (2002)
pWJ1165	YFP	Reid *et al*. (2002)
pWJ1350	RFP	Lisby *et al*. (2003)
EasyClone vectors for expression of genes for fluorescent proteins in *S. cerevisiae*		
pCfB393	pX-2-LoxP-KlURA3-TEF1::CFP	This study
pCfB394	pX-3-LoxP-KlLEU2-TEF1::RFP	This study
pCfB395	pX-4- LoxP-SpHiS5-TEF1::YFP	This study
Episomal vectors for expression of genes for fluorescent proteins in *S. cerevisiae*		
pCfB396	pESC-URA-USER-TEF1::CFP	This study
pCfB397	pESC-LEU-USER-TEF1::RFP	This study
pCfB398	pESC-HIS-USER-TEF1::YFP	This study
Plasmid contains a cassette for expression of GFP in *E. coli* (used as positive control in USER cloning)		
pCfB774	pmExpCtrl	Dr Hao Lao, DTU

**Figure 1:**
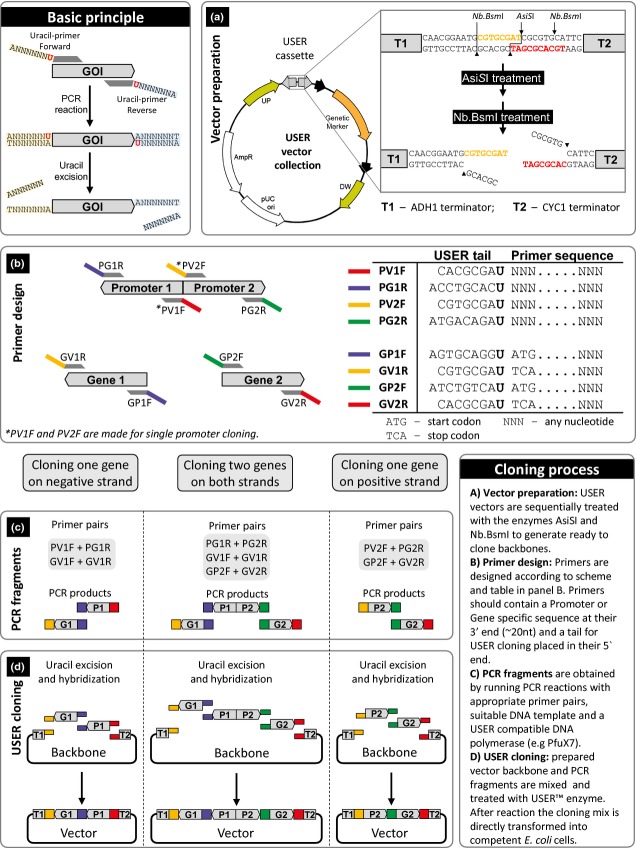
Overview of the procedure for cloning genes into EasyClone vectors. Detailed protocol can be found as Supplementary Material (Fig. S1).

Plasmids expressing fluorescent protein were constructed using USER cloning as previously described in Geu-Flores *et al*. (2007). Prior cloning, episomal and integrative vectors containing USER cassettes were digested with AsiSI and subsequently with the nicking endonuclease Nb.BsmI (Fig.1). Each batch of USER vector prepared for USER cloning (Supporting Information, Fig S1) was tested for the number of background transformants, that is, the number of transformants growing on selective plates but that do not carry a vector with the insert, and for the percentage of positive transformants, that is, the number of transformants which can grow on selective medium and which have received a vector with an insert. This experiment was carried as follows. A defined amount of prepared USER vector (*c*. 30 ng) was mixed with a PCR product bearing the green fluorescent protein encoding gene under the control of an *E. coli* promoter. This PCR product was generated using primers ID1493 and ID1494 and pCfB774 as template. Prepared USER vector and PCR product were mixed in a 1 : 3 vector to insert molar ratio. After USER reaction and transformation of chemically competent *E. coli* cells, cells were spread on LB_AMP_ and incubated for *c*. 18 h at 37 °C. The plate was directly analyzed under blue light excitation (bench top blue light table): the number of white colonies corresponds to the number of ‘background’ transformants, while the fluorescent colonies are the ‘positive’ transformants. Routinely at least 80–90% of the colonies were fluorescent.

The coding sequences for the genes encoding the three different fluorescence proteins and *TEF1* promoter were amplified by PCR using PfuX7 polymerase and primers listed in Table1. *CFP*, *RFP,* and *YFP* were obtained from appropriate plasmid templates pWJ1163, pWJ1350, and pWJ1165, respectively, and the promoter *TEF1* from genomic DNA of the CEN.PK113-11C strain. The promoter *TEF1* and cDNAs were cloned into previously linearized and tested expression vectors. A total of six constructs were produced: three designed to integrate on chromosome X and three to be expressed episomally. All constructed plasmids were validated by sequencing.

Yeast strain CEN.PK102-5B was transformed with different combinations of either episomal plasmids or linearized fragments for chromosomal integration by the lithium acetate transformation protocol (Gietz & Schiestl, 2007). Prior transformation, integrative plasmids were digested with NotI and the fragment containing the sequences for integration was purified from agarose gel. For each integrative fragment, we used 300–700 ng DNA for each transformation. For episomal plasmids, 200–400 ng DNA was used per transformation. The integration of the plasmids was verified by PCR analysis on yeast gDNA purified by the ZR Fungal/Bacterial DNA MiniPrep™ kit from Zymo Research using primers listed in Table1.

### Flow cytometry analysis

Transformants were grown o/n in SC-His-Leu-Ura, and 50 μL was used to inoculate 3 mL Delft medium in 24-deep-well plates, where the cells were grown at 30 °C with 300 r.p.m. agitation. When the cultures had reached mid-exponential phase, they were harvested and fixed with paraformaldehyde according to the following protocol. 1.5 mL samples were taken and immediately cooled in ice-water bath and subsequently centrifuged at 4 °C, 2000 ***g*** for 2 min. Supernatant was removed and pellet was resuspended in 200 μL of 2% paraformaldehyde. The mix was incubated on ice for 1 h and subsequently centrifuged at 4 °C, 2000 ***g*** for 2 min. Finally, the paraformaldehyde was removed and pellet was resuspended in 200 μL PBS. The fixed cells were stored at 4 °C until FACS analysis (maximum 1–2 days).

Cells were analyzed on a BD FACSAria equipped with three solid-state diode lasers: air-cooled Coherent™ Sapphire™ solid-state diode laser (488 nm, 100 mW), air-cooled Coherent™ Yellow Green laser (561 nm, 100 mW), and an air-cooled Coherent™ Deep Blue laser (445 nm, 50 mW). The following filters were used: FITC-A, PE-Cy5-A, and mCFP-A for the analysis of emission from yellow fluorescent proteins (YFP), red fluorescent proteins (RFP), and cyan fluorescent proteins (CFP), respectively. Compensation was performed according to the manufacturer's protocol (BD FACSAria II User's Guide).

Flow cytometry data sets were analyzed and interpreted by software packages derived from the open source platform of bioconductor (Gentleman *et al*., 2004). Outliers were removed by pregating on FSC and SSC data sets with the rule for outliers set at 90% quantile region. Cells were analyzed for their mean values, extracted as vectors, and plotted by the scatterplot3d function (Ligges & Maechler, 2003).

### Cre-LoxP-mediated selection marker loop out

Strains were transformed with pSH65 (EUROSCARF) harboring the *cre* gene under control of the *GAL10* promoter, and transformants were selected on YPD containing 10 μg mL^−1^ phleomycin (InvivoGen). Single colonies were picked and grown in YPD for 4–6 h, harvested by centrifugation and resuspended in YPG, where they were subsequently grown for another 12–16 h. Dilutions of the culture were then plated on YPD plates, and the emerging colonies were replica-plated on YPD, SC-Ura, SC-His, and SC-Leu to verify that all three markers had been looped out. Strains showing successful triple selection marker loop out were analyzed by flow cytometry as described above using SC-complete media as growth media.

## Results and discussion

**Figure 2:**
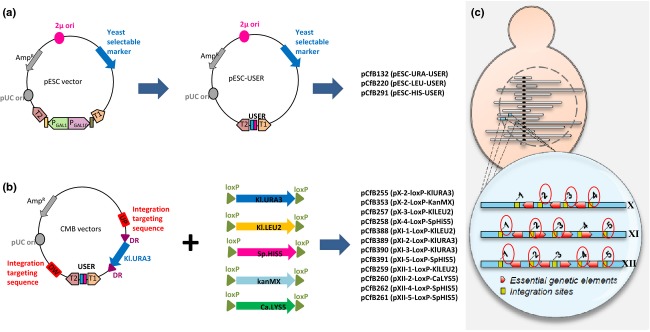
Plasmid construction. (a) Episomal vectors are based on the pESC vector (Agilent), where the multiple cloning sites and galactose-induced promoters were replaced by uracil excision-based cloning cassette. (b) Integrative vectors are based on the vectors described in Mikkelsen *et al*. (2012). The *URA3* selection cassette flanked by direct repeats was exchanged with the different selective markers indicated, all of which are flanked by *LoxP* sites allowing Cre-mediated marker loop out. (c) Integration sites were organized on chromosomes X, XI, and XII. All integration sites (yellow boxes) are separated by either genetic elements that are essential for growth or by genes essential for maintaining wild-type growth rates (red boxes). Integration sites encircled in red provide good level of gene expression, have minimum risk of spontaneous loop out or rearrangements, and do not impair growth.

To decrease the turnaround time in the metabolic engineering cycle, two sets of plasmids, one episomal and one integrative, were created (Fig.2). The episomal set was derived from a subset of the pESC plasmid series (Agilent). Specifically, the multiple cloning sites and *GAL1/GAL10* promoters were replaced by a uracil excision-based cloning cassette, AsiSI/Nb.BsmI (Hansen *et al*., 2011), hence making it USER cloning and USER fusion compatible, see Fig.1 and (Nour-Eldin *et al*., 2006; Geu-Flores *et al*., 2007).

The integrative vector set, which we named EasyClone, is based on the integrative plasmids from Mikkelsen *et al*., 2012 and also contains AsiSI/Nb.BsmI USER cassette. Specifically, we chose the vectors in the set, where the matching integration sites were shown to accept foreign DNA without affecting fitness of the strain and where gene expression was high (Mikkelsen *et al*., 2012). For these vectors, the *K. lactis URA3* selection cassette was substituted for one of five different selective markers (see Fig.2). To be able to reuse the introduced selection markers, the different markers are all flanked by *LoxP* sites, whereby the selection marker can be looped out by Cre recombinase-mediated recombination (Gueldener *et al*., 2002; Ito-Harashima & McCusker, 2004).

Both episomal and integrative plasmids contain two terminator sequences in opposite directions flanking the USER cloning cassette. This facilitates incorporation of two genes and a bi-directional promoter, while the option of incorporating only one single gene with one-directional promoter remains (Fig.1). The design of the cloning cassette ensures directional cloning. It also provides flexibility for the combination of different genes with different promoters using the same gene PCR fragment for any combination as long as the position of the gene is maintained, that is, Gene1 or Gene2. The different promoter fragments can be combined with any genes having the specified eight-nucleotide overhang, which allows for high-throughput cloning in a combinatorial setup.

**Figure 3:**
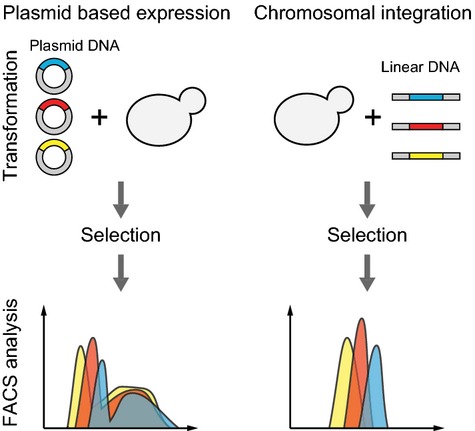
Experimental setup. *CFP*, *YFP*, and *RFP* were cloned into either episomal or integration vectors under the control of the strong *TEF1* promoter. *Saccharomyces cerevisiae* was transformed with either three episomal or three integration vectors followed by flow cytometric analysis for presence of the three fluorescent proteins.

To create a proof of concept for the plasmid set, an experiment was set up where the expression of three genes encoding three different fluorescent proteins from either episomal plasmids or from three integration sites in the genome was tested and compared (Fig.3). *CFP*, *YFP,* and *RFP* were cloned into pESC-USER and three integration plasmids, whereby six plasmids were constructed: pESC-CFP-URA, pESC-RFP-LEU, pESC-YFP-HIS, pX-2-CFP-LoxP-URA, pX-3-RFP-LoxP-LEU, and pX-4-YFP-LoxP-HIS. Strains were constructed harboring either the three pESC-xFP plasmids or the three integration xFP expression fragments.

The three integration fragments carrying the three fluorescent protein encoding genes were transformed into yeast in a single transformation event. From this triple transformation, 16 clones were tested for correct insertion by PCR. For seven clones, all the expected bands were seen on DNA electrophoresis, and all of these exhibited triple fluorescence from CFP, RFP, and YFP (results not shown). This showed that it is indeed possible to do triple integration in a targeted fashion with a relatively high success rate (44%).

**Figure 4:**
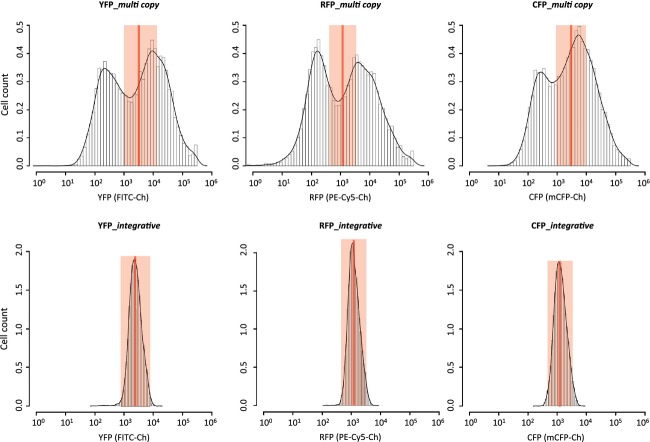
Flow cytometry on *Saccharomyces cerevisiae* strains co-expressing *YFP*, *RFP*, and *CFP* from either three episomal plasmids (top panel) or from triple integrations on the genome (bottom panel). The Log_10_ mean value ± 15% for each color is indicated with a deep red vertical line and a light red shading, respectively.

To test the individual production of the three fluorescent proteins in the two different strains containing the genes either on episomal 2μ plasmids or as triple genomic integrations, the fluorescence levels of single cells were analyzed by flow cytometry (Fig.4). Triple fluorescent protein production in strains containing the relevant genes as genomic integrations was much more uniform, as compared to strains where the genes were harbored on episomal plasmids. The mean levels of fluorescence were in the same range for the two expression systems, whereas the standard deviations for cells expressing the three fluorescent proteins from episomal plasmids were 4–5 times larger than for cells expressing from triple integrations (Table3).

**Table 3:** Log_10_ mean values with standard deviations for each fluorescence signal for cell producing CFP, RFP, and YFP

	Integration	Episomal plasmids
CFP	3.11 ± 0.21	3.40 ± 0.83
RFP	3.10 ± 0.20	3.30 ± 0.98
YFP	3.38 ± 0.22	3.41 ± 0.94

**Figure 5:**
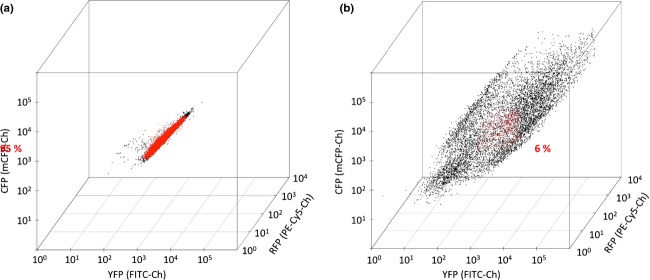
3D plot of the fluorescence levels of cells expressing *YFP*, *RFP*, and *CFP* from either triple genomic integrations (left box) or from episomal plasmids (right box). Each dot in the plot represents a cell with a certain fluorescence signal from YFP, RFP, and CFP on the *x-*, *y-*, and *z*- axes, respectively. Red dots represent cells having fluorescence intensities for all three fluorophores being within Log_10_ mean ± 15% for each color, and black dots represent cells with one or several fluorescence levels being outside of mentioned interval.

Next, we determined the levels of simultaneous production of the three fluorescent proteins in individual cells and plotted the data into three-dimensional plot (representing the levels of YFP, CFP, and RFP) (Fig.5). This analysis convincingly demonstrated that cells expressing the three genes from episomal plasmids are much more scattered throughout the whole three-dimensional space, whereas the cells with genomic integrations are in a much more defined space. As a measure for uniformity of protein production in the two systems, we defined that cells containing a fluorescent signal deviating from Log_10_ mean ± 15% for each color are identical for all three colors (highlighted in red on Fig.5). Based on this definition, only 6% of the cells harboring the episomal expression system contained identical levels of fluorescent proteins. In contrast, more than 95% of the cells were identical when the genes were integrated into the genome. This clearly demonstrates the advantage of the EasyClone plasmid set for the construction of complex pathways in yeast, as it is important to have stable and concomitant expression of all genes introduced in each cell to draw sensible conclusions.

For construction of large pathways or for repeated cycles of metabolic engineering, it is important that all markers used in a multiple integration experiment can be recycled. We therefore tested whether it would be possible to eliminate all three selection markers used for the integration of the genes encoding YFP, RFP, and CFP simultaneously. A strain expressing all three fluorescent proteins was transformed with a *cre*-containing plasmid and *cre* was subsequently induced by growing the transformant on galactose to allow for production of Cre recombinase. Ninety-six clones generated in this manner were tested for successful selection marker loop out and eight of these showed histidine, uracil, and leucine auxotrophy. All eight strains were retested for fluorescence and all showed fluorescent patterns, which were indistinguishable from the pattern produced by the parent strain (Fig. S2). The low level of *ura his leu* clones was most likely due to the proximity of the integration sites. The three integration sites were all on the same chromosome, which meant that there were 6 *LoxP* sites introduced within a fairly small genomic region of 42 kb. Hence, there was a risk of recombination between *LoxP* sites in two different integration sites with a lethal loss of an essential gene element to follow. Indeed, we obtained efficiencies above 90% for removal of selection markers int-egrated on different chromosomes (our unpublished results).

## Conclusions

In conclusion, we have shown that using EasyClone integrative vector set, it is possible to introduce up to three integration cassettes in *S. cerevisiae* genome simultaneously. Each integration cassette can be constructed to carry 1–2 genes. The selection markers used for the integration can be looped out simultaneously without the loss of the integrated genes. We also showed that expression of multiple genes from integrative cassettes leads to more homogeneous expression within the yeast population than expression from multiple episomal vectors. Combined with the fact that vector construction is based on highly efficient USER cloning, our system is well suited for the construction of cell factories containing multiple genetic modifications. The EasyClone vector set is available on request.

## Supporting Information

Fig S1 Overview of the USER cloning procedure.

Fig S2 FACS analysis of the cells before and after the triple selection marker loop out.
