# Supplementary material for: EasyClone: method for iterative chromosomal integration of multiple genes in Saccharomyces cerevisiae
Source: FEMS Yeast Res. 2013 Nov 18;14(2):238–48. doi: 10.1111/1567-1364.12118 (PMC4282123; doi:10.1111/1567-1364.12118)
Supplement: Fig S1 — Overview of the USER cloning procedure. [file fyr0014-0238-SD1.pdf]

Overview of the USER cloning procedure

Preparation of a batch of USER vector

20 µg of purified USER VECTOR
16 µL FastDigest AsiSI (ThermoScientific)
20 µL 10x FastDigest buffer (ThermoScientific)
Reaction volume 200 µL

↓ Digestion 37°C, 1h

Purification on column
(Typically use a Gel and PCR Clean up kit)

↓

The whole eluate
1 µL Nb.BsmI (New England Biolabs) / µg digested vector
Appropriate volume of Buffer 3.1 (New England Biolabs)

↓ Nicking 65°C, 1h

Purification of linear, nicked vector from agarose gel (Typically use a Gel and PCR Clean up kit)

↓

Quantification (ng DNA.µL^{-1})
Evaluation of background and positive control*

→ Storage (-20°C)

Collection of *Ready to use* USER VECTORS

Preparation of the inserts

PROMOTER Fragment*** GENE Fragment***

↓

PCR using PfuX7 (Nørholm, 2010)
For details on primer design, see Figure 1.

↓

Purification of products on agarose gel (Typically use a Gel and PCR Clean up kit)

↓

Quantification (ng DNA.µL^{-1})

→ Storage (-20°C)

Collection of *Ready to use* PROMOTER fragments and GENE fragments

USER cloning

~30 ng of prepared USER VECTOR**
x ng of prepared PROMOTER fragment**
y ng of prepared GENE fragment**
1 unit USER™ enzyme (New England Biolabs)
2 µL of PCR Buffer 10x
Reaction volume 20 µL

↓ USER cloning 37°C, 15min; 25°C, 15min

Transform *E. coli* chemically competent cells

↓

Spread transformed cells on selective medium

* for details, see Mat erials & Methods section
** for x and y: tipically use molar ratio vector :insert of 1:3 but this may require optimization (general guidelines in Nour-Eldin el al. 2006)
*** different combinations are possible , see Figure 1.
