# Supplementary figures and images for: EasyClone: method for iterative chromosomal integration of multiple genes in Saccharomyces cerevisiae
Source: FEMS Yeast Res. 2013 Nov 18;14(2):238–48. doi: 10.1111/1567-1364.12118 (PMC4282123; doi:10.1111/1567-1364.12118)

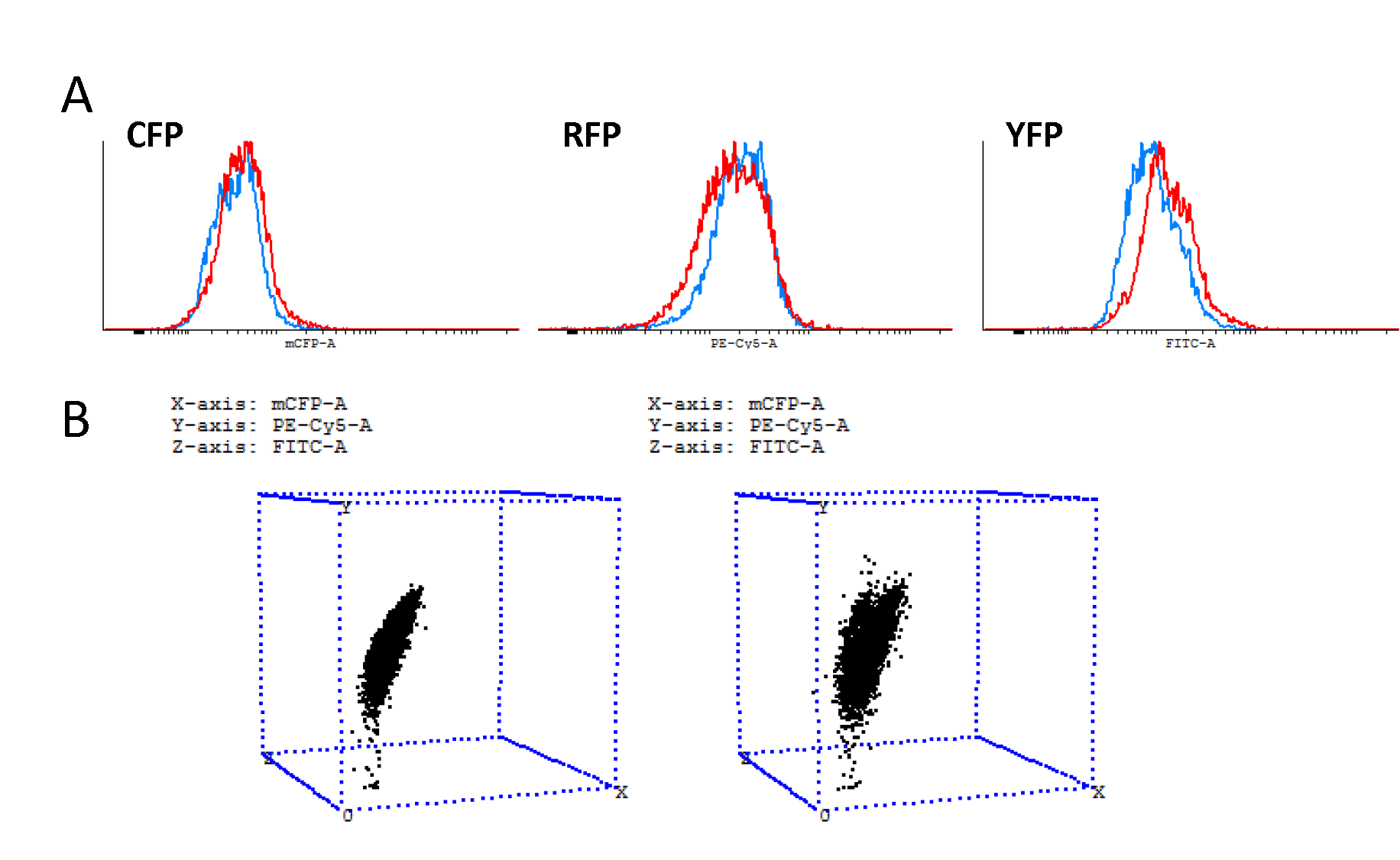

Supplement: Fig S2 — FACS analysis of the cells before and after the triple selection marker loop out. [file fyr0014-0238-SD3.tiff]
